# Opiate Sensitization Induces FosB/ΔFosB Expression in Prefrontal Cortical, Striatal and Amygdala Brain Regions

**DOI:** 10.1371/journal.pone.0023574

**Published:** 2011-08-23

**Authors:** Gary B. Kaplan, Kimberly A. Leite-Morris, WenYing Fan, Angela J. Young, Marsha D. Guy

**Affiliations:** 1 Mental Health Service, VA Boston Healthcare System, Boston, Massachusetts, United States of America; 2 Research Service, VA Boston Healthcare System, Boston, Massachusetts, United States of America; 3 Departments of Psychiatry and Pharmacology and Experimental Therapeutics, Boston University School of Medicine, Boston, Massachusetts, United States of America; University of Medicine and Dentistry of New Jersey, United States of America

## Abstract

Sensitization to the effects of drugs of abuse and associated stimuli contributes to drug craving, compulsive drug use, and relapse in addiction. Repeated opiate exposure produces behavioral sensitization that is hypothesized to result from neural plasticity in specific limbic, striatal and cortical systems. ΔFosB and FosB are members of the Fos family of transcription factors that are implicated in neural plasticity in addiction. This study examined the effects of intermittent morphine treatment, associated with motor sensitization, on FosB/ΔFosB levels using quantitative immunohistochemistry. Motor sensitization was tested in C57BL/6 mice that received six intermittent pre-treatments (on days 1, 3, 5, 8, 10, 12) with either subcutaneous morphine (10 mg/kg) or saline followed by a challenge injection of morphine or saline on day 16. Mice receiving repeated morphine injections demonstrated significant increases in locomotor activity on days 8, 10, and 12 of treatment (vs. day 1), consistent with development of locomotor sensitization. A morphine challenge on day 16 significantly increased locomotor activity of saline pre-treated mice and produced even larger increases in motor activity in the morphine pre-treated mice, consistent with the expression of opiate sensitization. Intermittent morphine pre-treatment on these six pre-treatment days produced a significant induction of FosB/ΔFosB, measured on day 16, in multiple brain regions including prelimbic (PL) and infralimbic (IL) cortex, nucleus accumbens (NAc) core, dorsomedial caudate-putamen (CPU), basolateral amygdala (BLA) and central nucleus of the amygdala (CNA) but not in a motor cortex control region. Opiate induced sensitization may develop via Fos/ΔFosB plasticity in motivational pathways (NAc), motor outputs (CPU), and associative learning (PL, IL, BLA) and stress pathways (CNA).

## Introduction

Sensitization is an addiction-related phenomenon that produces enduring enhancement of behavioral or locomotor responses after repeated and intermittent administration of psychostimulants and opiates [1]. Extensive investigations have shown that the repeated administration of psychostimulants such as cocaine, amphetamine, and nicotine result in persistent motor and neurochemical sensitization in animal models [2], [3]. Sensitization to drugs of abuse and associated stimuli is hypothesized to occur via plasticity in motor, motivational and cognitive neural systems and is thought to contribute to drug craving and relapse in addiction. Sensitization develops to the stimulant effects of opiates [4], [5] and to their conditioned rewarding properties, as measured by conditioned place preference [6], [7]. Sensitization develops to the conditioned aversive effects of withdrawal after repeated drug administration [8]. Thus, addiction can be viewed as a progressive phenomenon resulting partly from the sensitization of the stimulant and rewarding effects of drugs and from the conditioned aversive properties of drug withdrawal. Sensitization can last for days, weeks or months after cessation of drug exposure and is thought to contribute to relapse in addiction [9].

Mesocorticolimbic and nigrostriatal dopaminergic pathways are two major ascending circuits that regulate behavior, motor activity, reward, and reinforcement in the brain [10], [11], [12]. Mesocorticolimbic pathways originating from the ventral tegmental area innervate the medial prefrontal cortex, the ventral striatum (nucleus accumbens or NAc) and the amygdala while the nigrostriatal dopaminergic pathway originates from the substantia nigra and projects to the dorsal striatum (caudate putamen or CPU). The nigrostriatal dopaminergic pathway plays a key role in movement initiation, learning of motor patterns [3], [12] and drug-related habit learning [10], [11], [12]. The mesocorticolimbic dopaminergic circuit plays a key role in the locomotor stimulant, rewarding, and sensitizing properties of drugs of abuse [12], [13]. Dopaminergic projections to medial prefrontal cortex and amygdala are critical in the development of psychostimulant sensitization [3], [14], [15]. However, the mechanisms and neural pathways involved in opiate sensitization are not well understood.

Sensitization has been shown to produce plasticity in striatal pathways as measured by induction of transcription and translation of new proteins. Transcription factor ΔFosB, a truncated splice isoform of FosB from the immediate early-gene Fos family, has been considered to be critical in the regulation of brain dopaminergic systems following repeated drug administration. Because of this protein's stability, ΔFosB elevations are persistent in specific brain regions for long periods of time after their induction [16], [17], [18]. ΔFosB induction and expression are thought to be responsible for specific neuroplastic changes after chronic administration of drugs of abuse that include cocaine [19], [20], nicotine [21], ethanol [20], and Δ^9^-THC, the active compound in marijuana [20]. FosB and ΔFosB accumulate in a region-specific manner in the brain and are induced in the NAc and dorsal striatum (CPU) following drug administration. Overexpression of ΔFosB enhances sensitivity to the rewarding and locomotor-activating effects of cocaine [20], [22], [23], [24], [25], [26]. Additionally, ΔFosB mutant mice (vs. wild-type controls) showed increased motor responses to a single cocaine dose and enhanced conditioned place preference to a lower dose of cocaine [27]. Mutant mice, however, did not demonstrate further increases in activity after cocaine sensitization.

Studies have not yet examined relationships between neural FosB/ΔFosB expression and opiate sensitization in cortical or limbic brain regions. Different chronic opiate administration paradigms activate FosB/ΔFosB in accumbal [20], [28], [29] and cortical [20] regions but the associated behavioral effects of morphine treatment were not linked to such changes. In our syudy of overexpression of ΔFosB did increase the rewarding and withdrawal properties of repeated morphine treatment [26]. In another important study [30], the association between opiate sensitized motor behaviors and ΔFosB levels have been demonstrated in striatum and ventral pallidum brain regions. There are other diverse functional neural pathways that contribute to opiate sensitization including cognitive, motivational, motor and associative circuitry. It is critical to examine linkages between opiate sensitized behaviors and FosB/ΔFosB levels in a variety of relevant brain regions. We examined the ability of repeated and intermittent morphine administration to induce locomotor sensitization in C57BL/6 mice and then assayed FosB/ΔFosB immunoreactivity in cortical, striatal and amygdala regions using quantitative immunohistochemistry.

## Materials and Methods

### Animals and housing conditions

Male C57BL/6 mice weighing 24–26 g (Charles River Laboratories, NC) were used in all experiments and were chosen because of their demonstrated sensitivity to the behavioral effects of morphine stimulation. The animal facility was approved by the Association for the Assessment and Accreditation of Laboratory Animal Care (AAALAC). Mice were housed under standard vivarium conditions in a temperature and humidity controlled environment where they were allowed food and water *ad libitum*. Animals were kept under a 12∶12 h light-dark illumination cycle and acclimated to the colony environment for 7 days prior to all experiments. Drug treatments and behavioral monitoring began after 0700 h and ended before 1700 h. Animal use procedures were approved by the Veterans Affairs Medical Center Animal Care and Use Committee and were conducted in accordance with the NIH Guide for the Care and Use of Laboratory Animals.

### Locomotor activity and morphine sensitization treatment regimen

#### Open field activity test

Locomotor activity was measured with an automated infrared activity monitoring system from Med Associates (43.2 cm×43.2 cm; St. Albans, VT) containing 16 infrared beams in the horizontal plane. Activity boxes were placed in sound attenuation chambers to avoid most environmental sounds in the test room. The locomotor activity associated with horizontal locomotion was defined as the total distance traveled (cm).

#### Treatment regimen

A morphine dose of 10 mg/kg (s.c.) has been well-established to induce sensitization in previous studies [2], [4], [5], [6], [31]. This dose was also chosen because of findings that chronic morphine at a dose 10 mg/kg increased ΔFosB levels in the nucleus accumbens and ventral pallidum [30]. For both behavioral and neurochemical studies, mice were assigned to the repeated vehicle treatment or repeated morphine treatment groups (n = 8–16 per group). For behavioral comparisons, other mice were assigned to the vehicle pre-treatment group and received morphine challenge on day 16 or to morphine pre-treatment and received vehicle challenge on day 16. To determine the effect of intermittent morphine administration on the development or expression of behavioral sensitization, mice were pre-treated with either morphine (10 mg/kg, s.c.) or sterile saline (10 ml/kg, s.c.) injections once a day on days 1, 3, 5, 8, 10, and 12 (total of 6 administrations). All mice were allowed to acclimate to the activity testing boxes for 30 min prior to any treatment to minimize novelty effects. Locomotor activity, measured as the total distance traveled in centimeters, was monitored and recorded immediately after each injection at 15 min intervals for 180 min. To examine the expression of intermittent morphine-induced sensitization, mice received a single challenge injection of either morphine or saline on day 16 after a 3-day treatment-free period and the locomotor activity was subsequently examined as above. The anesthetic sodium pentobarbital (80 mg/kg, i.p.) was used to euthanize mice at the end of all experiments.

### FosB/ΔFosB immunohistochemistry

The effect of repeated morphine versus vehicle pre-treatments on FosB/ΔFosB induction in the brain was examined after vehicle injection and locomotor monitoring on day 16. Mice were anaesthetized and underwent intracardiac perfusion with ice-cold phosphate buffered saline (PBS) followed by 4% paraformaldehyde in 0.2 M phosphate buffer. Brains were removed quickly and post-fixed in a series: 4% paraformaldehyde, 12.5% sucrose in PBS, and 25% sucrose in PBS (24 h each). FosB/ΔFosB immunoreactivity was examined in free floating sections as described in a previous publication [31]. Brain regions studied were based on findings from other drug sensitization studies. Briefly, coronal sections (40 µm) from prelimbic cortex (PL), infralimbic cortex (IL), the NAc core (NAcC) and shell (NAcS), dorsal medial caudate-putamen (CPU), the central nucleus of amygdala (CNA) and the basolateral amygdala (BLA) regions were cut on a freezing cryostat. After blocking with 1.5% normal goat serum for 1 h, sections were incubated overnight at 4°C with the primary antibody, a rabbit polyclonal FosB antiserum (sc-48; Santa Cruz Biotechnology, CA, dilution 1∶750 in PBS containing 1.5% goat serum) that recognizes both FosB and isoforms of ΔFosB. Sections were then washed and incubated for 1 h at room temperature in secondary antibody, biotinylated goat anti-rabbit IgG (Santa Cruz Biotechnology, Santa Cruz, CA, dilution 1∶2000 in PBS). FosB/ΔFosB immunoreactivity was visualized by the biotin-streptavidin technique (ABC Staining System; Santa Cruz Biotechnology, Inc., Santa Cruz, CA) using 3, 3′diaminobenzidine (ImmunoPure Metal Enhanced DAB; Pierce, Rockford, IL) as the chromagen.

### Quantitation of FosB/ΔFosB immunoreactivity

Changes in FosB/ΔFosB immunoreactivity were measured in sections from prefrontal cortices (PL, IL), striatal (NAcC and NAcS, CPU), and amygdala (CNA and BLA) brain regions. Quantitative measurement was performed using a computer-assisted image analysis system, consisting of an Olympus BX51 bright field microscope interfaced with a color digital camera (MicroFire; Optronics, Goletta, CA ), and a computer with Image-Pro Plus image processing & analysis software (Version 6.3; Media Cybernetics, Silver Spring, USA). Images were obtained at 10× magnification and were averaged from right and left hemispheres in each subject. FosB/ΔFosB-positive nuclei were considered to be signals showing grayscale contrast levels over 125 units (total possible range was 0–255) in order to eliminate very lightly stained nuclei constitutively present in the brain. The individuals performing the analyses were blinded to treatment conditions.

### Statistical analysis

For pre-treatment studies (days 1–12), two-way ANOVA was used to analyze the influence of morphine vs. saline pre-treatments (as the between-group factors) and the day of treatment (as the within-group factor) as well as the treatment by time interaction on locomotor activity. For locomotor activity on day 16 of treatment, one-way ANOVA was used to analyze the influence of treatment groups. Bonferroni tests were used to determine differences between individual treatment groups for locomotor activity. T-test comparisons were used to examine treatment effects (vehicle pre-treatment vs morphine pre-treatment) on FosB/ΔFosB levels in each brain region. *P*<0.05 was considered statistically significant. Outlier analyses were performed for treatment groups and a data outlier was defined if its standard score is ±2.5 standard deviations from the mean [32]. There were only three data point outliers identified; these were not included in analyses.

## Results

**Figure 1:**
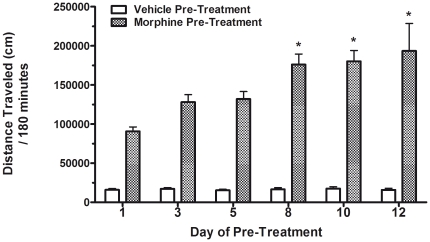
Intermittent morphine administration results in the development of locomotor sensitization in C57BL/6 mice. Mice were given either a saline (10 mg/ml) or morphine (10 mg/kg) injection on days 1, 3, 5, 8, 10, 12 and a locomotor activity test was conducted and recorded immediately after each injection for 180 min. Data are recorded as total distance traveled for 180 min and shown as total distance traveled (cm) ± S.E.M in each treatment group (n = 8–16 mice per group). *P<0.05 in comparison to the morphine treated group's first day of testing.

Figure 1 illustrates the motor effects of intermittent morphine administration given on days 1, 3, 5, 8, 10, and 12 of pre-treatment, as measured by the total distance traveled over 180 min. A two-way ANOVA demonstrated significant effects of treatment (vehicle vs. morphine) group (F_1, 232_ = 235.4; *P*<0.0001), treatment day (F_5, 232_ = 3.47; *P*<0.01) and treatment group×day interaction (F_5, 232_ = 3.39; *P*<0.0001). Post-hoc testing revealed that on days 8, 10, and 12 of treatment, the morphine treatment groups showed significant differences in locomotor responses compared with day 1 of treatment. Thus, repeated morphine pre-treatment produced increasing motor stimulant effects within four to six treatments resulting in the development of locomotor sensitization in this model.

**Figure 2:**
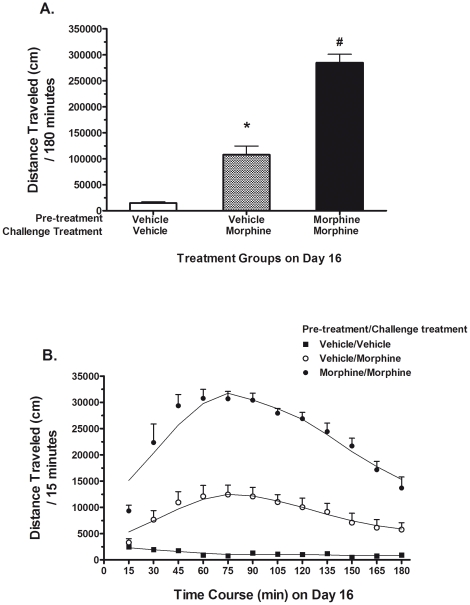
Intermittent morphine administration results in the expression of locomotor sensitization in C57BL/6 mice. A) Mice were given either a saline or morphine (10 mg/kg, s.c.) injection on days 1, 3, 5, 8, 10, and 12. On the challenge day (day 16), mice were treatment-free for a three day period and then received a single challenge dose of morphine (10 mg/kg) or saline. Locomotor activity tests were performed for 180 min immediately after drug injections and data are recorded as total distance traveled (cm) ± S.E.M in each treatment group (n = 8 mice per group). *P<0.05 vs. vehicle pre-treatment/vehicle challenge group; # *P*<0.05 vs. vehicle pre-treatment/morphine challenge group. B) The time course of opiate sensitization (measured in 15 min intervals) is demonstrated on challenge day after three treatment-free days from intermittent morphine administration on day 16.

Figure 2A shows the locomotor activity values for three different treatment groups: vehicle pre-treatment/vehicle challenge, vehicle pre-treatment/morphine challenge, and morphine pre-treatment/morphine challenge. On the challenge day, the vehicle pre-treatment group receiving morphine challenge showed a 5-fold increase in mean distance traveled (vs. vehicle/vehicle controls). The morphine pre-treatment group receiving morphine challenge showed a 12-fold increase in motor activity (vs. vehicle pre-treatment/morphine challenge). These results show a model for the expression of morphine sensitization. One-way ANOVA shows significant differences in activity levels for these three treatment groups (F_2, 23_ = 101.9; *P*<0.0001). Figure 2B illustrates the time course (at 15 min intervals) of locomotor response to a morphine challenge injection over 180 min on day 16. On challenge day, mice receiving morphine showed maximum activity levels reached at 60 to 90 min and demonstrate increased stimulant effects of morphine challenge after morphine pre-treatment. These results suggest that after six pre-treatments, morphine administration on day 16 induces the expression of morphine sensitization.

**Figure 3:**
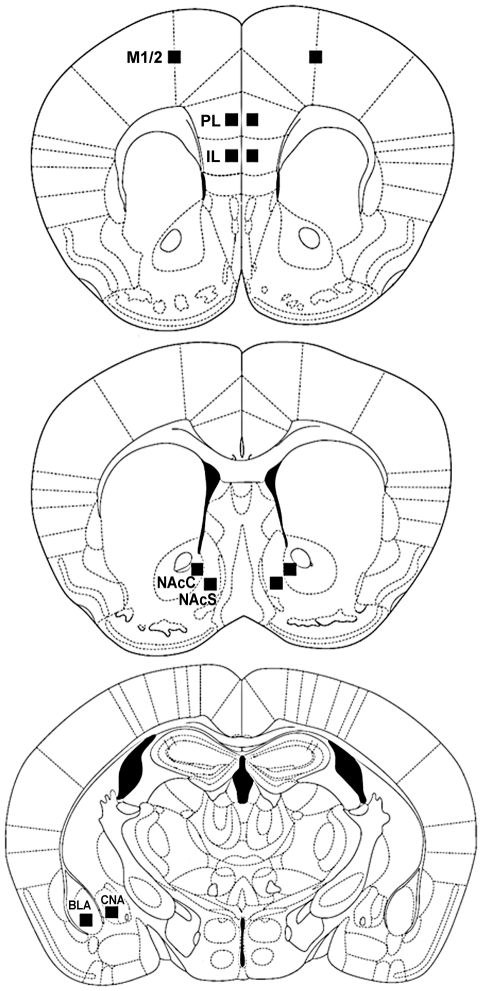
Schematic diagrams of coronal sections of mice brain illustrating where FosB/ΔFosB immunoreactivity was quantified in the following regions: prelimbic cortex (PL), infralimbic cortex (IL), motor cortex (M1/2), nucleus accumbens core (NAcC), nucleus accumbens shell (NAcS), caudate-putamen (CPU), central nucleus of amygdala (CNA) and basolateral amygdala (BLA). The black squares correspond to a fixed area 200 µm×200 µm in size.

Figure 3 shows schematic diagrams of coronal sections of mouse brains and illustrates locations of FosB/ΔFosB immunoreactivity quantitation in PL, IL, NAcC, NAcS, CPU, and BLA regions and a control motor region (M1/2). The black squares correspond to a fixed area 200 µm×200 µm. In the PL, the square was placed equal in the center of PL (1.70 to 1.42 mm). In the IL, the square was placed equal in the center of IL (1.70 to 1.42 mm). In the M1/2, the square was placed the centrally at the border between M1 and M2 (1.70 to 1.42 mm). In the NAcC, the left vertical length of the square was placed medial to the anterior commissure and the top horizontal width was placed above the anterior commissure (1.54 to 0.98 mm). In the NAcS, the right vertical length of the square was placed lateral to the major islands of Calleja and the top horizontal width was placed with the dorsal edge at equal height with the anterior commissure (1.54 to 0.98 mm). In the CPU, the rectangle was placed at the top of the lateral ventricle (1.54 to 0.98 mm). In the amygdala subregions, the rectangles were placed inside the CNA (−1.06 to −1.34 mm) and inside the BLA (−1.06 to −1.34 mm).

**Figure 4:**
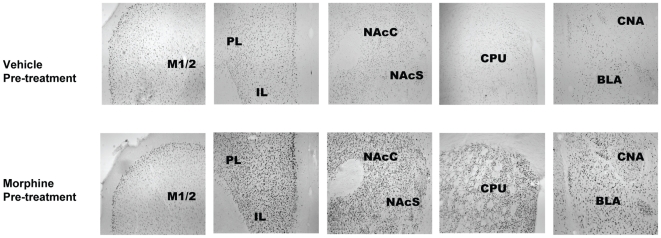
ΔFosB/FosB expression was determined by immunohistochemistry using anti-FosB/ΔFosB polyclonal antibody in multiple sections. These representative photomicrographs (10-fold magnification) demonstrate FosB/ΔFosB immunoreactive nuclei in PL, IL, M1/2, NAcC, NAcS, CPU, CNA and BLA brain regions. They were collected from vehicle and morphine pre-treatment treatment groups following vehicle injection and motor activity testing on day 16. The landmarks include: ACa, anterior commissure, and LV, lateral ventricle.

**Figure 5:**
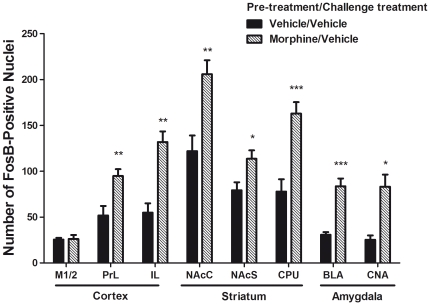
Quantitative analyses of FosB/ΔFosB immunoreactivity from subjects from the vehicle and morphine pre-treatment groups following vehicle injection and motor activity testing on day 16. The quantification of the ΔFosB/FosB expression was determined by immunohistochemistry from multiple sections in each of the following brain regions: PL, IL, M1/2, NAcC, NAcS, CPU, CNA and BLA. Data are shown as mean ± S.E.M of n = 4–8 mice from each treatment group. *P<0.05, **P<0.01, and ***P<0.001 vs. vehicle pre-treatment group.

FosB and ΔFosB are neural transcription factors which measure adaptive changes following morphine treatment and were assayed by quantitative immunohistochemistry. Figure 4 shows representative photomicrographs of FosB/ΔFosB-positive nuclei in brain regions which include PL, IL, M1/2, NAcC, NAcS, CPU, CNA and BLA. FosB/ΔFosB-positive nuclei were quantified inside a field of 200 µm×200 µm within each brain region of interest. Figure 5 illustrates mean counts of FosB/ΔFosB-positive nuclei in brains from the morphine pre-treatment group and vehicle pre-treatment groups that were harvested from mice following vehicle injection and motor activity testing on day 16. For each treatment group, mean counts from bilateral regions were averaged and analyzed via ANOVA procedures. T-test comparisons demonstrated significant effects of repeated morphine treatment on FosB/ΔFosB levels in each of the following brain regions: PL (t = 3.1; *P*<0.01), IL (t = 3.7; *P*<0.005), NAcC (t = 3.2; *P*<0.005), CPU (t = 4.0; *P*<0.001), BLA (t = 3.9; *P*<0.001) and CNA (t = 2.8; *P*<0.05) and no differences in M1/2.

## Discussion

In summary, repeated and intermittent morphine injections produced progressive increases in motor activity. In morphine pre-treated mice, a morphine injection on challenge day (day 16) produced more than a two-fold increase in locomotor behavior compared to vehicle pre-treated mice (receiving the same morphine challenge). Morphine sensitized mice demonstrated associated increases in FosB/ΔFosB immunoreactivity in PL, IL, NAcC, NAcS, CPU, CNA, and BLA regions.

Opiate sensitization has been characterized by an increased sensitivity enhancement to the effects of opiates after prolonged exposure [1]. Its development and expression depend on dose and duration of drug administration as well as the withdrawal time from the last exposure to the drug [33], [30]. The intermittent morphine treatment regimen in our study produced progressively larger increases in the locomotor response to morphine consistent with previous results and the dose administered is comparable to previously demonstrated ranges [4], [5], [30], [31], [33], [34], [35].

Transcription factor ΔFosB is a highly stable protein induced in the brain's reward regions by chronic exposure to many drugs of abuse and is an important regulator of both dopaminergic and glutamatergic transmission in the brain [17], [26], [28], [30], [36], [37]. It is well documented that this transcription factor accumulates in the brain as a result of exposure to a variety of stimuli, including drugs of abuse [16], [38], [39]. Previous studies have shown that ΔFosB accumulates with repeated exposure to drugs of abuse and remains at high levels in the brain for weeks [18], [40], [41] representing a potential mechanism underlying the long-lasting neuroadaptation. It has been shown that ΔFosB is induced after repeated exposures to drugs of abuse [17], [20], [38] and in cocaine sensitization [19]. Over-expression of ΔFosB in nucleus accumbens and dorsal striatum increased locomotor and rewarding responses to both cocaine and morphine [9], [22], [26]. One previous study had demonstrated FosB/ΔFosB induction in accumbal and ventral pallidal regions in opiate sensitization [30] but it did not examine such effects in cortical, dorsal striatal, and limbic brain regions as demonstrated in this report.

This study demonstrates the importance of FosB transcription factors and neuroadaptive responses in cortical and amygdala brain regions in opiate sensitization. It highlights that diverse pathways contribute to opiate sensitization including cognitive, motivational, motor and associative neural circuits. Mueller and Unterwald [28] demonstrated that repeated and increasing doses of morphine, but not acute morphine, produced increases in ΔFosB-immunoreactivity in the NAc, CPU and PL. Zachariou and colleagues [26] demonstrated that ΔFosB over-expression in the NAc enhanced sensitivity to morphine reward and dependence. McDaid and coworkers [30] demonstrated that morphine-induced behavioral sensitization was associated with elevated accumbal and pallidal ΔFosB levels following a 3-day withdrawal that returned to baseline in 14 days. Our results are consistent with these findings and all studies highlight the importance of accumbal FosB/ΔFosB induction as a plasticity marker in opiate addiction.

This study presents new findings in which opiate sensitization produces neuroadaptive effects in executive (PL, IL), motivational (NAc), motor (CPU), and associative learning (BLA) pathways. The PFC is responsible for executive function, decision-making, and the implementation of goal-directed actions. Subregions of the PFC include the PL, which guides response initiation and the IL which mediates response inhibition; both regions guide actions and outcomes. The PL and IL may serve as on-off mechanisms in both conditioned drug and fear responses [42], [43]. The BLA processes emotion-related drug stimuli and mediates approach or avoidance behavior. The NAc connects input from limbic sites related to drug reward to behavioral output through the ventral pallidum. The CNA is a major output area for stress responses.

A role for mesocorticolimbic dopamine neurons has been previously demonstrated in opiate sensitization. Repeated and intermittent morphine treatment produced an enhancement of motor activity associated with increases in mesolimbic dopamine transmission [2], [44], [45]. Because the NAc receives glutamatergic projections from prefrontal cortex and amygdala regions [3], it functions as an interface for dopamine-glutamate interactions. The NAc regulates both emotional and behavioral responses and is implicated in the expression of behavioral sensitization to opiates [3], [14]. It is likely that the enhanced locomotor sensitivity induced by intermittent morphine treatment in the present study is accompanied by long-lasting synaptic adaptations in these dopaminergic and glutamatergic neurons [3], [46] that appears to be produced by ΔFosB mechanisms.

However, comparisons between different sensitization studies is challenging because dopaminergic systems are responsive to various parameters of morphine treatment including morphine dose, time between withdrawal and challenge treatment, as well as genetic background of animals and species differences [45], [47], [48]. A limitation of this study is that the antibody used measures both FosB and ΔFosB proteins, so future studies can examine these transcription factors separately in these various regions. Since diverse pathways are activated during opiate sensitization, future studies might also examine how plasticity in motivational and associative circuits along with motor outputs may contribute individually and interactively to opiate sensitization responses. Future translational research is needed to examine behavioral and neurobiological mechanisms of enduring hyper-responsivity to drug sensitization associated structural plasticity which underlies drug craving and relapse. In summary, this study suggests that opiate sensitization produces the induction of FosB/ΔFos plasticity in executive (PL, IL), motivational (NAc), motor (CPU), and associative learning (BLA) pathways.
